# Intramedullary reaming modality for management of postoperative long bone infection: a prospective randomized controlled trial in 44 patients

**DOI:** 10.1186/s13037-019-0215-3

**Published:** 2019-12-02

**Authors:** Carlos Augusto Finelli, Fernando Baldy dos Reis, Helio Alvachian Fernandes, Adriana Dell’Aquila, Rogério Carvalho, Natalia Miki, Carlos Franciozi, Rene Abdalla, Mauro José Costa Salles

**Affiliations:** 1 Department of Orthopedics and Traumatology, (DOT/UNIFESP), Escola Paulista de Medicina, Universidade Federal de São Paulo, Rua Napoleão de Barros 715, São Paulo, SP 04024-002 Brazil; 2 Santa Casa de São Paulo School of Medical Sciences, São Paulo, SP Brazil

**Keywords:** Intramedullary nail, Infection, Osteomyelitis, Reaming, Irrigation, Aspiration

## Abstract

**Background:**

Studies addressing the management of intramedullary infection are mainly retrospective and with a limited number of cases. Reaming can be performed using either conventional reaming or using the reamer/irrigator/aspirator (RIA) system. Until now there have been no comparative prospective studies between these two methods. We aimed to compare the efficacy of RIA with conventional reaming followed by insertion of antibiotic-loaded cement, for the treatment of intramedullary nail infection of long bones. We assessed the rate of remission between groups after two-year follow-up and identified microorganisms using tissue cultures and sonication of explanted intramedullary nail (IMN).

**Methods:**

A noninferiority, randomized clinical trial was carried out between August 2013 and August 2015 involving 44 patients of whom a locked IMN implant of the femur and/or tibia was retrieved and who all met the clinical and radiological criteria for IMN-associated osteomyelitis. Patients were randomized into two groups: RIA alone versus conventional reaming followed by antibiotic-loaded cement insertion. Both groups also underwent six-weeks of antibiotic treatment according to the results of the antibiogram. Patients were evaluated after 1, 3, 6, 12 and 24 months for radiological and clinical follow-up.

**Results:**

After 24 months, the rate of infection remission was similar between the two groups, 87% in the RIA group and 95.5% in the conventional reaming group (*p* = 0.60). Among four patients who had recurrence of infection, the time to reappearance of symptoms varied from 20 days to twenty-two months. *Staphylococcus aureus* and coagulase-negative Staphylococci were isolated in 23 (40.4%) and 13 (22.9%) patients, respectively. Interestingly, we identified 20% (9/45) of polymicrobial infection.

**Conclusion:**

This study concludes that the RIA system alone, is noninferior to conventional reaming followed by antibiotic cement spacer in the treatment of IMN infection. However, RIA shows greater efficacy in the collection of infected medullary bone tissue, mainly in cases of infected retrograde nail of the femur.

**Trial registration:**

ISRCTN82233198. Retroactively registered on July 29, 2019.

## Background

Intramedullary nailing is the preferred fixation method for the treatment of femur and tibia shaft fractures. With an increase in trauma volumes resulting from road traffic accidents, low and middle-income countries will face a proportional increase in infections associated with an intramedullary implant [1]. In high-income countries, the rate of long bone intramedullary nail infection varies from 1 to 2%. This rate is substantially increased in low- and middle-income countries [1, 2]. Principle of infection management includes surgical debridement to remove devitalized soft tissue and planktonic microorganisms. Nailing explantation is an important step to reduce biofilm load, followed by medullary reaming, irrigation, soft tissue coverage and anti-biofilm specific antibiotic therapy [3–6].

To date, conventional reaming remains the preferred method to achieve debridement of the infected medullary canal, combined with antibiotic loaded-cement spacer implantation and an optional second surgery for its removal [3–6]. However, pitfalls of this technique include bone overheating, which may result in thermal osteonecrosis, an uncertainty of complete eradication of infected tissue, propagation of the infection throughout the entire medullary canal or systemically, and pulmonary thromboembolism [7]. The Reamer / Irrigator / Aspirator (RIA) system, (Synthes®, Inc. West Chester, Philadelphia, PA, USA) was developed to reduce fatty embolism and to decrease the systemic inflammatory process prior to nail insertion [8, 9]. Its use and indication were rapidly expanded to harvest of autologous bone graft in addition to the treatment of femoral medullary canal infection [10, 11]. Recent studies suggest that RIA is a safe, effective and promising procedure for intramedullary infection treatment [7, 12].

To date, no randomized controlled studies comparing RIA versus conventional reaming combined with antibiotic cement nail in patients presenting with intramedullary infection have been performed [4, 5, 7, 9, 11, 13]. Our aim was to compare the two above-mentioned treatment options focusing on the two-year remission rates, by performing a prospective randomized study, investigating the treatment of intramedullary infection of femur and tibia. We hypothesized that no difference in the rate of two-year remission would be identified in patients treated with RIA or conventional reaming and antibiotic nail following a diagnosis of intramedullary infection.

## Methods

### Study design and population

Our study is a prospective, randomized, controlled, single-blinded clinical trial which included 46 consecutive patients with clinically suspected intramedullary infection who underwent nailing explantation and surgical debridement followed by either conventional reaming or RIA of the presumed infected medullary canal. The study was performed in a large orthopedic trauma center hospital between August 2013 and December 2015. All patients included in the study were appropriately consented for their inclusion in the research study. This study was approved prior to initiation, by the Research and Ethics Committee of the institution, under the number 354.934, 08/16/2013.

The inclusion criteria for the study were patients of 18 years and over; those with previous tibia or femur intramedullary locking nail fixation who met the definitive clinical and radiological diagnosis of osseous infection. Patients with diaphyseal diameter < 10 mm, those with previous infection of the affected bone and patients with HIV or chronic renal failure were excluded from the study. Patients presenting intramedullary infection were assessed at the Trauma unit and the outpatient setting by the infection disease specialist and recruited after signed consent. Allocation of patients to the treatment groups took place at the time of anesthesia within the operative room. Then, randomization to conventional reaming or RIA was set up in a simple randomization procedure (1:1 ratio) with sealed opaque envelopes, sequentially numbered. The CONSORT guidelines were followed throughout the study [14]. We defined IMNI according to the criteria of the Center for Disease Control and Prevention (CDC) / National Healthcare Safety Network (NHSN) guidelines (https://www.cdc.gov/nhsn/pdfs/pscmanual/pcsmanual_current.pdf).

### Clinical and microbiological assessment

We assessed and analyzed variables regarding patient demographics and comorbidities; injury-association data, including anatomical site of fracture, mechanism of trauma and Gustilo type of fracture; surgery-related factors, including open reduction and internal fixation or two-stage fixation with temporary external fixator; microbiological findings and antibiotic therapy; and the patient outcome.

Patients who underwent surgical treatment received 1 g of intravenous vancomycin starting soon after tissue samples were obtained and IMN were retrieved for sonication. Empirical therapy with vancomycin was maintained until the definitive results of tissue and sonication fluid cultures were obtained. Antibiotic therapy was further adjusted based on the susceptibility tests provided. Patients were kept in hospital to complete at least 2 weeks of intravenous antibiotic treatment, and then discharged for a six-week course of oral antibiotics. During surgical procedure, up to five soft tissue and bone samples were collected, then placed into identified sterile recipients and processed for microbiology and histopathology. Nails were aseptically removed, placed into sterilized (autoclave at a max temperature of 121 °C for 15 min or plasma sterilization) sealed lock-lock® polyethylene containers, to which 200 ml of Ringer solution was added and labeled with patient’s data. Upon arrival at the laboratory, the containers were vortexed and submitted to sonication as previously described [15, 16].

### Surgical procedure

In the conventional reaming group, a cortical window was made in the distal section of the bone, from one distal locking hole to another, using a 4.5 mm drill. It was intended to expel the debridement material during reaming. Reaming was performed using consecutive reamers, increasing in size up to 1.5 mm wider in diameter than that of the removed nail. This step was alternated with the introduction of a 0.9% saline solution, approximately 3 l, using a 60 ml syringe. The collected material was separated into five samples, stored in sterile containers and sent for microbiological analysis. Soon after, an antibiotic loaded-cement spacer was custom-made using a 3 mm guide wire and a chest tube by mixing 40 g of cement with 2 g of vancomycin [17]. Once the cement polymerized, the chest tube was incised using a scalpel blade and the antibiotic loaded-cement spacer was ready to use. The spacer was inserted into the medullary canal and removed between 15 and 20 days later. Once removed, the antibiotic spacer was placed in a sealed container (lock-lock®) with Ringer solution (200 ml) and sent for sonication [15, 16].

In the RIA group, no cortical bone window was performed. The medullary canal was measured using a radiopaque gauge and the size of the reamer was chosen according to the diameter, using a minimum of 1.5 mm larger diameter than the removed nail. Reaming was initiated, and irrigation was performed with 3 l of 0.9% saline solution until the collector was filled. The content of the collector was separated into five samples and sent for microbial analysis. No antibiotic-loaded cement spacer was inserted.

### Follow-up and study endpoints

The follow-up visits were performed in the outpatient setting at 30 days, three, six, 12 and 24 months after discharge from the study infection hospitalization. Clinical, inflammatory marker tests such as C-reactive protein (CRP), and *erythrocyte sedimentation rate* (ESR) and radiological test were assessed. A telephone interview was also performed, 24 months after intramedullary infection surgery. Patients were evaluated by the Visual Analog Scale (EVA) scale in relation to preoperative pain and postoperative follow-up [18]. Primary outcome was the intramedullary infection two-year remission rate, which was performed using all randomized patients that completed at least one-year of follow-up in the per-protocol (PP) analysis. Patients were considered in remission when there was an absence of clinical, laboratory and radiological signs of infection, assessed in the last medical consultation (at least 12 months follow-up). Cases that did not require re-operation or further antibiotic administration for the same site of infection were also considered to be in remission [19, 20].

### Treatment failure or recurrence

Treatment failure or recurrence was defined as infection at the same surgical site, which had previously been brought under control and required surgery and /or a second course of intravenous antibiotic therapy [3, 20, 21]. Diagnosis of recurrence was based upon the occurrence of at least one of the following: (1) wound requiring additional surgery more than 2 weeks after the last of the initial debridement for infection and randomization; (2) culture-positive recurrence of infection before bony union as evidenced by persistently elevated or progressively increasing CRP and ESR in the context of recurrent wound drainage and no history of inflammatory arthritis; (3) infection-induced joint erosion that requires arthrodesis or amputation to eradicate infection.

### Statistical analysis

The exploratory data analysis included mean, median, standard deviation and variation for continuous variables, frequency and proportion for categorical variables. The normal distribution of continuous variables was analyzed by asymmetry, kurtosis and Kolmogorov-Smirnov test. Comparison between groups was performed by Student’s t-test for continuous variables and Pearson’s chi-square test or Fisher’s exact test for categorical variables. Comparison of the pain scale between related groups was performed by Wilcoxon signed-rank test. Cumulative disease-free survival analysis for both groups (RIA and conventional reaming), was carried out using Kaplan-Meier method and compare with the *log rank* test. Statistical analysis was performed using the IBM-SPSS Statistics version 24 software (IBM Corporation, NY, USA). All tests were two-tailed and values of *P* <  0.05 were considered significant.

## Results

During the study we included 46 patients, of which two patients did not meet the inclusion criteria and were excluded from this study. In one case the patient’s chart showed treatment of previous infection prior to intramedullary osteosynthesis. In the other case HIV contamination was diagnosed. Forty-four patients were analyzed (45 implants as one patient presented ipsilateral infection of the femur and tibia at different time point), mechanism of trauma was mainly due to high energy trauma (93.2%). Demography and clinical characteristics of the study population are presented in Table 1.

**Table 1:** Characteristics of the 44 patients

Features	
Age, years (SD)	34.8 ± 10.8
Gender, *n* (%)	
Male	38 (86)
Diabetes (%)	1 (2.3)
Smoking (%)	6 (14)
Type of accident, *n* (%)	
Motorcycle collision	28 (63.6)
Overwhelmed	7 (16)
Car collision	4 (9.1)
Fall from height	3 (6.8)
Injury by firearm	2 (4.5)
Bone*, *n* (%)	
Tibia	29 (64.4)
Femur	16 (35.6)
Exposed fracture*, *n* (%)	33 (73.3)
External osteosynthesis* *n* (%)	31 (68.9)

Continuous variables are described in mean ± standard deviation; variables are described in number (proportion). * Analysis on 45 implants

Reaming with RIA system was performed in 23 cases, while conventional reaming was performed in 22. The comparative analysis showed no statistical difference between the groups regarding demographics, comorbidities, type of trauma and first-step damage control orthopedics. Data are shown in Table 2.

**Table 2:** Comparative analysis between groups according to intervention RIA and CR

	RIA (*n* = 23)	CR (*n* = 22)	*P* value
Age,years	33.61 ± 9.6	36.05 ± 12	0.46
Gender, *n* (%)			
Male	19 (82.6)	19 (86.4)	1.00
Female	4 (17.4)	3 (13.6)	
Diabetes (%)			
Yes	0 (0)	1 (4.5)	0.48
No	23 (100)	21 (95.5)	
Smoking (%)			
Yes	4 (17.4)	2 (9.1)	0.66
No	19 (82.6)	20 (90.9)	
Type of accident, *n* (%)			
Collision or trampling	21 (91.3)	19 (86.4)	0.66
Other	2 (8.7)	3 (13.6)	
Bone*, *n* (%)			
Tibia	14 (60.9)	15 (68.2)	0.60
Femur	9 (39.1)	7 (31.8)	
Open fracture*, *n* (%)			
Yes	19 (82.6)	14 (63.6)	0.15
No	4 (17.4)	8 (36.4)	
External osteosynthesis* *n* (%)			
Yes	18 (78.3)	13 (59.1)	0.16
No	5 (21.7)	9 (40.9)	

Continuous variables are described in mean ± standard deviation; categorical variables are described in number (proportion). *RIA* Reamer-irrigator-aspirator, *CR* Conventional reaming followed by spacer cement with antibiotic

Upon analyzing pain, according to patients’ complaints before and after nail removal, a significant improvement was seen in both groups (*p* <  0.001). Using the visual numerical scale for pain, the mean intensity was rated at seven points prior to nail removal and zero points after (Table 3).

**Table 3:** Analysis of pain, according to numerical visual scale, before and after intervention

	Before	After	*P* value
Pain*			
All patients	7 (0–10)	0 (0–5)	< 0,001
RIA group	7 (1–10)	0 (0–5)	< 0,001
CR group	1 (0–10)	0 (0–5)	< 0,001

*Results described in median (min - max). *RIA* Reamer-irrigator-aspirator, *CR* Conventional reaming followed by spacer cement with antibiotic

**Fig. 1:**
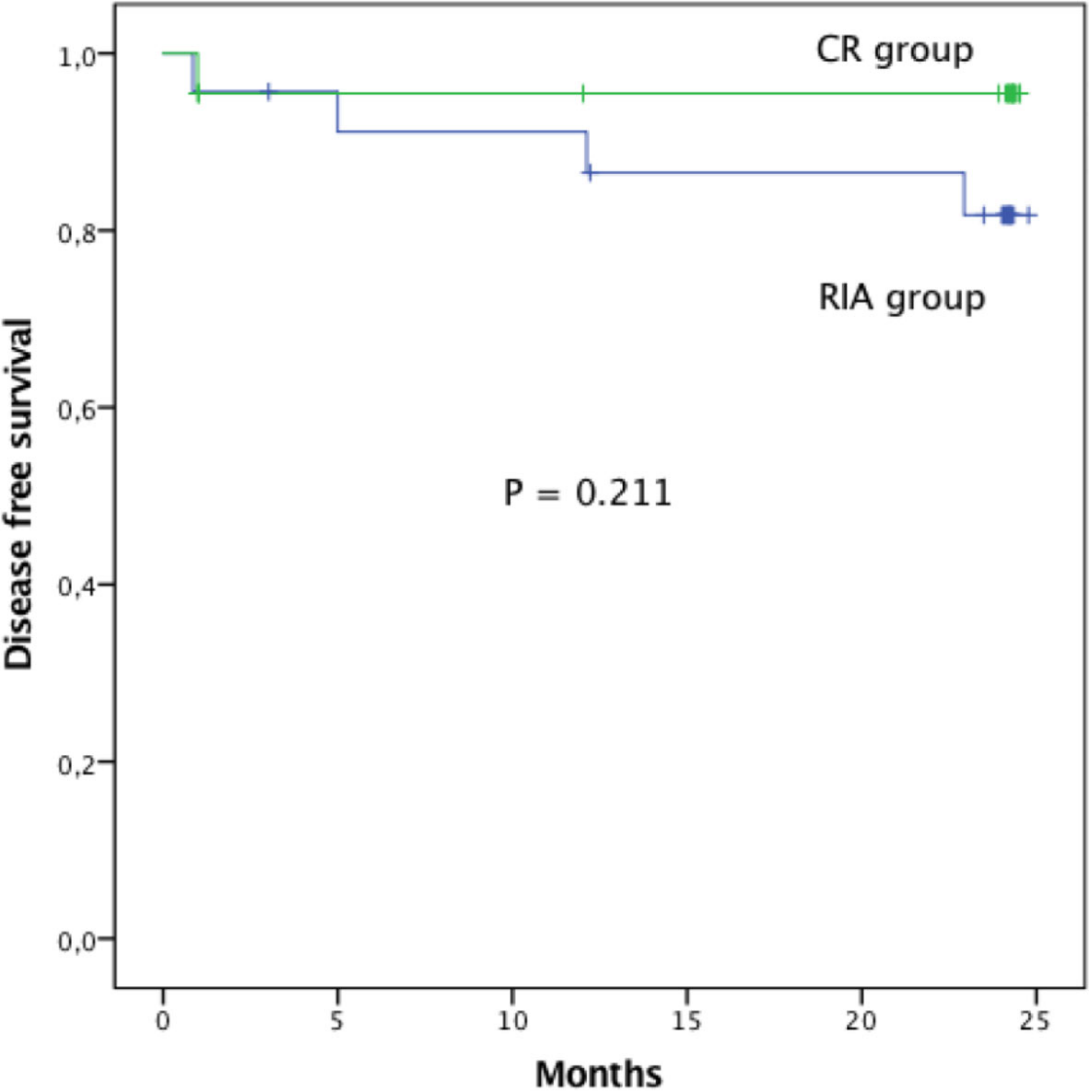
Kaplan-Meier estimate of disease-free survival rate showing no difference between two-year survival rate for conventional reaming and RIA

With regards to primary outcome until the completion of two-year follow-up, four patients (9.1%) presented signs and symptoms of relapse, of which three patients were from the RIA group and only one patient from the conventional reaming group. The 2-year cumulative osteomyelitis-free survival rate of conventional reaming and RIA was 95.5 and 81.7%, respectively. The Kaplan-Meier curve was plotted showing no difference in the 2-year cumulative osteomyelitis-free survival rate among conventional reaming and RIA groups (*p* = 0.211, *Log rank* test), (Fig. 1).

All cases of recurrence presented Gustilo grade III-A or III-B open fracture as initial trauma with segmental bone loss and significant soft tissue injury. Three patients had previously been treated with an external fixation device as a damage control strategy.

The most common bacteria isolated from tissue cultures and sonication of retrieved implants were *S. aureus*, followed by coagulase-negative Staphylococci as seen in Table 4. Polymicrobial infection yielded in nine patients (20%).

**Table 4:** Microbial profile resulting from tissue and sonication cultures

*S. aureus*	23 (40,4%)
CNS*	13 (22,9%)
*Enterococcus sp*	5 (9,0%)
*Enterobacter sp*	4 (7,0%)
*Pseudomonas aeruginosa*	3 (5,3%0
*S. pyogenes*	3 (5,3%)
*Klebsiella* spp*.*	2 (3,3%)
*S. agalactiae*	1 (1,7%)
*Providencia* spp*.*	1 (1,7%)
*Serratia* spp*.*	1 (1,7%)
*Proteus sp.*	1 (1,7%)

*CNS Coagulase-negative Staphylococci, including *S. epidermidis*

Categorical variables are described in number (proportion)

Regarding the outcome of the comparative costing study, the RIA group represented 1.83% less cost than the CR group, which includes the cost of antibiotics, cements and the need for second hospitalization for removal of cement rods.

## Discussion

The most relevant finding of this study was the similarity of performance between the RIA system and conventional reaming followed by an antibiotic-loaded spacer in the treatment of intramedullary infection, after 24 months follow-up. There was no statistically significant difference between the two groups with regards to the primary endpoint, when the bone infection was associated with the presence of intramedullary nail. To our knowledge this is the first randomized prospective study comparing the efficacy of conventional reaming and RIA for the treatment of femur and tibia intramedullary infection [7, 9, 22]. Nevertheless, we acknowledged that a higher number of open and severe fractures as well as those previously treated with external fixator were allocated in with RIA system group despite of the randomization, which may have hindered the final results. It is possible that RIA system may show superiority over CR while performing studies using higher number of patients.

Furthermore, to be able to increase the microbial diagnosis of intramedullary infection we carried out a thorough microbiological evaluation by using tissue cultures, sonication of the retrieved nails and the antibiotic-loaded spacers [15, 16]. Sonication has been described as a highly accurate diagnostic procedure in implant-associated infections and the results of this case series prompt the widening of the scope for the diagnosis of intramedullary infection [15, 23]. In this study, sonication presented high sensitivity in the diagnosis of infection of explanted nails (data no shown).

Pitfalls were encountered for the collection of debris from reaming in one patient with a retrograde nail in the femur, who was randomly selected for the conventional reaming. In this case, access to the proximal locking hole in the anterior thigh was hindered by the muscle thickness and the decubitus position, so a large curette was used to remove the intramedullary material from the muscle envelope after reaming. Despite this, it was not as effective in the collection of tissue as it was in the tibia, using conventional reaming. It is worth pointing out that in two cases of conventional reaming with anterograde nails in the femur, even after cutting a window in the bone, the amount of debris expelled from reaming was inadequate, despite having injected saline solution into the proximal point of entrance. It was therefore necessary the use of a catheter inserted at the entrance in order to allow the saline solution to be expelled through the distal window of the bone. In fact, there is a possibility of overheating during intramedullary reaming, which reduces the effectiveness of extracting the content of the medullary canal [11]. This condition may explain the reason why the quantity of tissue expelled through the window in the distal segment of the affected bone was much less in the conventional reaming than the RIA group, which varied from 60 to 80 cc. As the RIA system uses interchangeable and disposable heads, the reaming together with continuous irrigation decreases the adverse effect of overheating and increases the amount of infected intramedullary tissue extracted [11, 24, 25].

Preliminary reports regarding treatment of infection related to IMN in long bones have documented different strategies for intramedullary infection, upon which two-stage procedures had been more commonly applied [4]. Some orthopedic surgeons prioritize bony union as the main part of treatment and advocate retaining the implant with surgical cleansing and debridement of devitalized tissue followed by suppressive intravenous antibiotic therapy. Conversely, others consider the eradication of the infectious process to be the most critical stage of the treatment, and the removal of the nail to be crucial for the removal of biofilm, followed by intramedullary reaming, debridement of the soft tissue, local and systemic antibiotic therapy, and insertion of a new IMN in cases of non-union [4, 26]. Despite satisfactory results on these publications, they were biased due to their retrospective manner, small sample size and the lack of detailed standardization of diagnostic methods and treatment protocols [4]. Moreover, the treatment of choice for intramedullary infection with conventional reaming followed by antibiotic cement spacer is a relatively recent method, and still needs further prospective investigation. Other investigators managed to demonstrate high long-term disease-free survival rate of patients with intramedullary infection treated conventional reaming and antibiotic-loaded spacers [5, 12, 22]. However, they performed either retrospective studies or case series with small sample size [4, 13].

The first published results of the RIA system being applied for the treatment of osteomyelitis was in 2007 that showed no recurrence in a six-month follow-up of 11 post-trauma infected patients [9]. Likewise, RIA studies applied as a surgical strategy for treating osteomyelitis following IMN are of low-quality evidence due to its retrospective fashion or the description of case series [12]. Nevertheless, unlike conventional reaming, the RIA system allows for reaming with constant irrigation and simultaneous aspiration, thus it has been deemed to reduce the risk of dissemination of infected material through the medullary canal and adjacent circulatory structures [11]. In our study it was clearly seen that in cases of conventional reaming, especially in the femur, biological elements from reaming were spread to the soft tissue through the distal bone window. In one case of infected retrograde femoral implant, the infected reamed tissue spread to the proximal anterior thigh muscle at the level of the anterior bone window and propagated, in a smaller quantity, into the knee articulation during exchange of reamer heads.

The cement-loaded spacers enable the release of high doses of antibiotic in the medullary canal, optimizing the control of infection in local tissue and minimizing the side effects of systemic antibiotic therapy [27]. However, after the period of antibiotic elution, the cement can act as substrata for bacterial growth and therefore the formation of biofilm [27]. This aspect was confirmed in this study, as the cement spacers retrieved that underwent sonication yielded microorganisms in most of our cases. We argue that, this may be one the reasons for recurrence when dealing with intramedullary infection, when the treatment of choice is a two-stage procedure, in which the cement spacer is left in the intramedullary cavity for a longer period. On the other hand, intramedullary infection has been successfully treated through an adequate reaming of the medullary canal, preferably using the RIA system, which does not require a second invasive surgical procedure for the removal of the antibiotic cement spacer. Although RIA system is a relatively new procedure, it has been regarded as the treatment of choice in many orthopedic centers of excellence in the developed world [7].

One the limitations of this study was the lack of Tscherme, Oesten classification (1982) for the soft tissue lesion in the initial trauma surgery. We were unable to access the patients’ charts from other institutions, as many patients with chronic intramedullary infection were later referred to our specialized center. Cierny and Mader classification for osteomyelitis was also not possible to be provided. Nevertheless, in the four cases of recurrence, all patients presented Gustilo grade 3 open fractures, along with bone loss and accentuated damage of the soft tissue. We also acknowledge the low number of patients randomized for each group, as well as the lack of other groups for comparison, for example RIA followed by antibiotic cement spacer as studied before [12]. However, we performed a randomized clinical trial in which the long-term two-year follow up was completed and enabled us to draw adequate conclusions.

## Conclusion

This study concludes that the RIA system alone was as effective as conventional reaming followed by antibiotic cement spacer in the treatment of intramedullary infection. Despite similar results regarding the rates of infection remission, RIA system was more effective to collect large amount of infected medullary bone tissue. In addition, among patients with femoral retrograde infected nail, it seems to be indispensable due to the minimal propagation of infected tissue into the knee and also to prevent the need to perform an anterior proximal cortical bone window. Although, antibiotic-loaded spacer following the reaming with RIA system was not applied, we currently recommend it for the treatment of intramedullary infection.

## Data Availability

The datasets used and/or analysed during the current study are available from the corresponding author on reasonable request.

## Abbreviations

ASA: American Society of Anesthesiologists
CDC/NHSN: Center for Diseases Control and Prevention/National Health Care Safety Network
CI: Confidence interval
CNS: Coagulase-negative Staphylococci
CRP: C-reactive protein
ESR: Erythrocyte sedimentation rate
EVA: Visual Analog Scale
HR: Hazard ratio
IMN: Intramedullary nail
MRSA: Methicillin-resistant *Staphylococcus aureus*
*MSSA*: Methicillin-sensitive *S. aureus*
PP: Per-protocol;
RIA: Reamer/irrigator/aspirator system
